# Dynamic characterization of breast cancer response to neoadjuvant therapy using biophysical metrics of spatial proliferation

**DOI:** 10.1038/s41598-022-15801-7

**Published:** 2022-07-09

**Authors:** Haley J. Bowers, Emily Douglas, Katherine Ansley, Alexandra Thomas, Jared A. Weis

**Affiliations:** 1 Department of Biomedical Engineering, Wake Forest School of Medicine, 575 N. Patterson Ave., Suite 530, Winston-Salem, NC 27101 USA; 2 Virginia Tech-Wake Forest University School of Biomedical Engineering and Sciences, Blacksburg, USA; 3 Atrium Health Wake Forest Baptist Comprehensive Cancer Center, Winston-Salem, USA; 4 Section of Hematology and Oncology, Department of Internal Medicine, Atrium Health Wake Forest Baptist, Winston-Salem, USA

**Keywords:** Computational models, Data processing, Predictive medicine, Breast cancer, Cancer imaging, Cancer therapy

## Abstract

Current tools to assess breast cancer response to neoadjuvant chemotherapy cannot reliably predict disease eradication, which if possible, could allow early cessation of therapy. In this work, we assessed the ability of an image data-driven mathematical modeling approach for dynamic characterization of breast cancer response to neoadjuvant therapy. We retrospectively analyzed patients enrolled in the I-SPY 2 TRIAL at the Atrium Health Wake Forest Baptist Comprehensive Cancer Center. Patients enrolled on the study received four MR imaging examinations during neoadjuvant therapy with acquisitions at baseline (T_0_), 3-weeks/early-treatment (T_1_), 12-weeks/mid-treatment (T_2_), and completion of therapy prior to surgery (T_3_). We use a biophysical mathematical model of tumor growth to generate spatial estimates of tumor proliferation to characterize the dynamics of treatment response. Using histogram summary metrics to quantify estimated tumor proliferation maps, we found strong correlation of mathematical model-estimated tumor proliferation with residual cancer burden, with Pearson correlation coefficients ranging from 0.88 and 0.97 between T_0_ and T_2_, representing a significant improvement from conventional assessment methods of change in mean apparent diffusion coefficient and functional tumor volume. This data shows the significant promise of imaging-based biophysical mathematical modeling methods for dynamic characterization of patient-specific response to neoadjuvant therapy with correlation to residual disease outcomes.

## Introduction

The current standard of care for locally-advanced and early-stage high-risk breast cancer includes pre-surgical neoadjuvant therapy (NAT) which includes a pre-determined regimen with pre-determined doses and cycles of anti-neoplastic therapy^1^. The goal of NAT is to reduce the tumor burden for better surgical outcome, increase the likelihood of breast conservation surgery, treat micrometastases, and avoid axillary lymph node (ALN) dissection^2^. Post-NAT pathologic staging systems play an important role in personalizing subsequent therapeutic interventions, including planning surgery and adjuvant therapy, as they have been shown to be predictive of recurrence risk and overall survival for this patient group. There are two main pathologic staging systems based on surgical pathology: pathological complete response (pCR) and residual cancer burden (RCB). pCR is a binary metric that indicates the presence or absence of residual tumor in the breast and/or ALN at surgical resection following the conclusion of NAT. RCB is a continuous metric with calculation based on components of tumor size, tumor cellularity, and ALN involvement and has been shown to identify patients who are at the highest risk for early recurrence^3,4^. Importantly, patients who have no residual tumor burden in the breast or axilla (pCR or RCB = 0) at the conclusion of NAT experience increased event-free and overall survival outcomes, occurring in about 20% of patients who undergo NAT across all molecular subtypes^5^. Conversely, patients who have residual disease at the conclusion of neoadjuvant therapy (non-pCR or RCB > 0) are at increased risk of early recurrence and death^6,7^. Unfortunately, at the individual patient level, conventional therapy response assessment tools are insufficient to determine disease eradication and allow for cessation of systemic pre-operative treatment.

Current conventional assessment tools for therapy response consists of physical examination and/or conventional morphometric breast imaging to assess changes in tumor size/volume. These assessment methods are not sensitive enough to accurately evaluate treatment response as they are influenced by the presence of fibroglandular tissue, post-therapy fibrosis, loss of palpability after treatment, and are susceptible to observer biases^8^. Magnetic resonance (MR) imaging is the most accurate imaging modality for assessment of tumor response in the NAT setting, but use is often limited to high risk breast cancer patients for initial staging and presurgical evaluation of response to NAT^9,10^. Currently, non-invasive imaging assessment of treatment response remains a challenging clinical problem with a lack of validated and approved tools for clinical translation^11^. MR imaging radiological response is conventionally assessed using volumetric measurements used to coarsely define tumor response, stabilization, or progression. While monitoring these morphologic changes has shown some utility in describing the final status of the tumor^12^, this fails to describe real-time response to therapy or the underlying mechanistic phenotypic biophysics governing tumor response, resistance, and heterogeneity. MR imaging has been proposed to have a role in early response assessment, and studies are evaluating the usage of MR imaging in early NAT response prediction^13,14^. Emerging quantitative MR methods have been established to measure biophysical tumor properties, with theoretical advantages over conventional assessment methods. Utilization of quantitative MR imaging modalities, such as dynamic contrast enhanced (DCE) MR and diffusion-weighted (DW) MR, have the potential to enable mechanistic biophysical assessment of response, improving upon basic morphological measurements. While quantitative MR imaging methods may allow for biophysical assessment, the overall assessment of treatment response remains limited with no current early-response, imaging-based biomarkers that have been validated to predict long-term outcomes and become standard of care in the clinical setting. The current lack of standardization of early and interval MR imaging throughout NAT, along with the existing coarse response assessment tools that lack mechanistic interpretation, limits opportunities for effective interventional therapeutic decisions during therapy. Development of new patient-specific comprehensive dynamic biophysical assessment tools may offer the opportunity to optimize therapeutic regimens based on observed response.

Clinical trial designs are utilizing quantitative imaging throughout the course of NAT^15–17^. For example, the multisite I-SPY TRIALs (Investigation of Serial Studies to Predict Your Therapeutic Response through Imaging and Molecular Analysis) is an ongoing phase II trial for women with high-risk breast cancer, focusing on testing new therapies coupled with imaging and tissue biomarker analysis^18–20^. Due to the growing interest in adaptive therapy regimens to optimize patient-specific therapy based on observed radiological response^21–23^, there is a need for clinically-validated imaging biomarkers to dynamically characterize the mechanistic patient response to NAT. We^24–26^ and others^27–30^ have shown the potential in using mechanistic computational modeling to interpret medical imaging data to predict patient-specific response to NAT in breast cancer. In previous work, we developed a mechanically-coupled reaction–diffusion model capable of parameterizing biophysical parameters of response from before and after one cycle of neoadjuvant therapy to predict the tumor burden at the end of NAT and achieved an area under the curve (AUC) for pCR prediction of 0.87 using the early imaging time point. Prediction of pCR based on early response assessment may help identify patients who may benefit from treatment de-escalation, but it leaves no guidance for the 70–80% of patients who do not achieve pCR. By establishing new methods to quantitatively characterize the dynamic biophysics of patient-specific therapeutic response, we aim to identify biomarkers to aid in clinical decision support throughout the NAT regimen to tailor therapy.

In this study, we examine an extension of our previously-developed methods to characterize dynamic response throughout the course of NAT by coupling our model-based imaging analysis methods with evaluation time points that harmonize within the I-SPY 2 TRIAL study design. The purpose of this work is to explore a proof-of-concept framework to provide greater insight to breast cancer NAT dynamic response and investigate associations of model-based characterizations of imaging data with residual cancer burden. Our approach interprets changes in serial MR quantitative imaging data using mechanistic biophysical mathematical modeling to characterize dynamic changes that occur over the course of NAT. This framework estimates phenotypic biophysical parameters of global cellular diffusive motility and spatial cellular proliferation rate. The goal of this study is to establish a proof-of-concept protocol for characterizing changes in MR imaging data using spatial estimates of tumor proliferation to enable accurate predictions of residual tumor burden.

## Methods

### Study cohort

This retrospective imaging analysis study is based on the review of breast cancer patients enrolled in I-SPY 2^18^ at the Atrium Health Wake Forest Baptist Comprehensive Cancer Center between April 2019 and July 2021 and was approved by the Institutional Review Board of Wake Forest Baptist Medical Center. All experiments were performed in accordance with relevant guidelines and regulations. Informed consent was obtained from all patients under the original data acquisition study and a waiver of informed consent was approved by the Institutional Review Board of Wake Forest Baptist Medical Center for this data re-analysis study. During the study period, 10 patients were enrolled in the study, 2 patients withdrew participation, and 2 patients were excluded due to image quality (aberrant positioning during image acquisition) or registration failure. 6 patients (median age, 50; range 44–58) with completed imaging data from I-SPY 2 were analyzed for all time points. Patient characteristics are included in Table 1. Images were acquired at baseline (T_0_), after 3 cycles of therapy (T_1_), mid-treatment (T_2_), and at the conclusion of NAT prior to surgery (T_3_). The median time between T_0_ and T_1_, T_1_ and T_2_, and T_2_ and T_3_ were 29, 67, and 76 days, respectively. The median time between the final imaging time point and surgery was 17 days. Patients were randomized to an experimental drug arm or to a control arm (standard of care) in accordance with the parent I-SPY 2 design.

**Table 1:** Patient characteristics for analyzed cohort.

Patient #	Age (years)	Tumor type	Tumor grade	Experimental arm	ALN status	RCB index
1	46	ER + /PR +	IB	Durvalumab + Olaparib	Negative	1.94
2	48	HER2 +	IIIA	Pertuzumab + Trastuzumab	Negative	0
3	50	ER + /PR +	IIB	SD-101 + Pembrolizumab	Negative	1.48
4	44	TNBC	IIIB	SD-101 + Pembrolizumab	Positive	3.32
5	54	ER + /PR +	IIA	Control (Paclitaxel only)	Positive	3.15
6	58	ER + /PR +	IIIA	Cemiplimab + REGN3767	Negative	2.60

**Figure 1:**
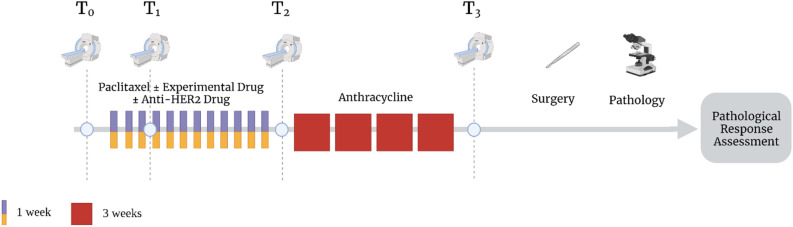
Schematic of imaging and therapy timeline. Patients undergo imaging prior to the start of neoadjuvant therapy, after three weeks of therapy, at the conclusion of the paclitaxel portion of therapy, and at the completion of therapy prior to surgery. After surgery, pathological response is determined based on pathological analysis of the resected tumor. Created with BioRender.com.

Figure 1 shows the study timeline whereby patients received 12 weekly cycles of paclitaxel (control) or in combination with one of the experimental agents, followed by 4 cycles of anthracycline-cyclophosphamide prior to surgical resection. Patients classified as HER2-positive also received anti-HER2 agents. Pathological analysis of the resected surgical sample is performed following surgery to determine pathological response with RCB parameters recorded.

## Imaging data acquisition

MRI was performed using a Siemens 3 T SKYRA scanner with a double-breast coil for all patients. Dynamic contrast enhanced MRI (DCE-MRI) utilized an acquisition matrix of 448 × 448 × 176 (both breasts) over an axial FOV of 360 mm × 360 mm with a slice thickness of 1 mm, and one single acquisition. Dynamic scans used a flip angle of 10˚. A catheter was placed within an antecubital vein and delivered gadolinium-based contrast at an injection rate of 2 cc/s (dose was dependent on patient weight) with a 20-cc saline flush. DCE-MRI acquisition was performed once pre-contrast and then six times after contrast injection using identical sequences, lasting for at least 8 min after contrast injection. DW-MRI was acquired with a single-shot spin echo imaging sequence in three orthogonal diffusion encoding directions, with b-values of 0, 100, 600, and 800 s/mm^2^, FOV of 250 mm × 360 mm. The DW-MRIs consisted of 40 axial slices with slice thickness of 5 mm, repetition time of 7000 ms, echo time of 74 ms, and flip angle of 90°. During imaging subjects were breathing freely with no gating.

### Imaging data analysis

Serial DW-MRI and DCE-MRI data obtained throughout the course of NAT were used to generate spatial estimates of tumor cell proliferation rates in response to therapy using a previously developed image data-driven biophysical mathematical modeling methodology^24–26^. DWI-MRI data were aligned to DCE-MRI data through scanner offset correction and pixel spacing interpolation. DCE-MRI and DWI-MRI data were then longitudinally co-registered across all imaging time points with rigid registration using FLIRT^31–33^ followed by non-rigid registration using DRAMMS^34^ using default registration parameters. Central-slice images through the midpoint of the tumor were extracted and used for subsequent analysis. DCE-MRI imaging data was used to create a tumor region-of-interest for each time point using initial manual segmentation with refinement based on voxels that satisfy a signal intensity threshold increase of 80% between the pre-contrast and the first post-contrast image^24,35^. DW-MRI data sets are fit to Eq. (1) to return the apparent diffusion coefficient (ADC) values for each voxel^36^:

1 $$ADC=\frac{\sum_{i=x,y,z}\mathrm{ln}\left(\frac{{S}_{0}}{{S}_{i}}\right)/{b}_{i}}{3},$$

where *i* describes the direction of diffusion weighting and *b*_*i*_ describes the total diffusion weighting imparted to the sample. *S*_*0*_ is the signal intensity in the absence of diffusion gradients, and *S*_*i*_ is the intensity in the presence of the diffusion-sensitizing gradient. Spatiotemporal cellularity, *N*($\overline{x }$,*t*) was estimated using Eq. (2) with ADC data for voxels satisfying the DCE-MRI threshold criteria of 80% enhancement^35^:

2 $$N\left(\overline{x },t\right)= \theta \left(\frac{{ADC}_{w}-ADC(\overline{x },t)}{{ADC}_{w}-{ADC}_{min}}\right),$$

where $\theta$ describes the cellular carrying capacity, a geometric constraint on the total number of tumor cells in a voxel, calculated as the ratio of the imaging voxel volume to the assumed tumor cell volume, assuming spherical tumor cells with a packing density of 0.7405 and a nominal tumor cell radius of 10 microns (tumor cell volume of 4189 μm^3^)^37^. ${ADC}_{w}$ is the ADC of free water at 37 °C (3e−3 mm^2^/s), $ADC(\overline{x },t)$ is the ADC value at each position in the image space, and ${ADC}_{min}$ is the minimum ADC measured within the tumor for each patient, corresponding to the voxel with the largest number of cells^36,38^.

As introduced in prior work^24^, the set of coupled, partial differential equations governing the clinical tumor growth model is shown in Eqs. (3)–(5):

3 $$\frac{\partial N(\overline{x },t)}{\partial t}= \nabla \cdot \left(D\nabla N\left(\overline{x },t\right)\right)+k(\overline{x })N(\overline{x },t)\left(1- \frac{N(\overline{x },t)}{\theta }\right)$$

4 $$D={D}_{0}{e}^{-\gamma {\sigma }_{VM}(\overline{x },t)}$$

5 $$ \nabla \cdot G\nabla \mathop{u}\limits^{\rightharpoonup} + \nabla \frac{G}{1 - 2v}\left( {\nabla \cdot \mathop{u}\limits^{\rightharpoonup} } \right) - \lambda \nabla N\left( {\overline{x},t} \right) = 0 $$

Equation (3) describes the spatiotemporal rate of change in cell number as the sum of random cell diffusion and logistic growth. Equation (4) links the apparent cell diffusion, *D*, to the surrounding tissue mechanics. ${\sigma }_{VM}$ is distortional (von Mises) stress, $\gamma$ is an empirically derived coupling constant, and ${D}_{0}$ is tumor cell diffusion in the absence of external stress^39^. Equation (5) is linear elastic, isotropic mechanical equilibrium exposed to an external expansive force based on tumor cell number changes as well as the empirically derived coupling constant, $\lambda$. $G$ represents the shear modulus defined as *G* = *E*/2(1 + *v*) where *E* represents Young’s modulus, and $v$ is Poisson’s ratio which is assumed as 0.45. $u$ is a vector which describes tissue displacement in response to tumor cell growth. Finite element meshes were constructed for each patient and were composed of three-node triangular elements with an average edge length of 1.5 mm. The mesh was then discretized into regions with an average of 5 elements per region and average area of 3.25 mm^2^ for subsequent model-based spatial property reconstruction using k-means clustering based on Euclidian distance. Temporal resolution was assigned at *Δt* = 1 day.

**Figure 2:**
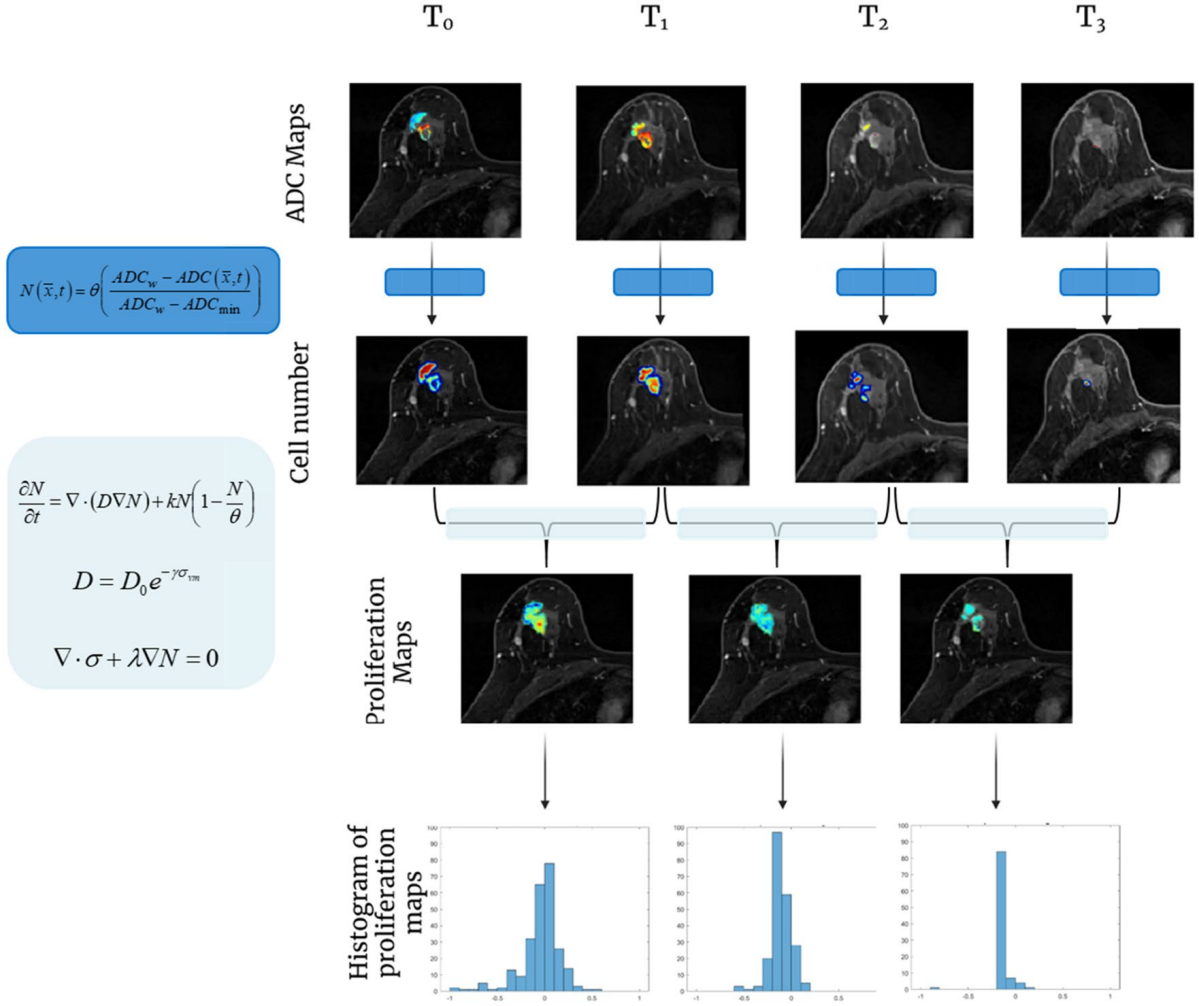
Schematic of the model-based methodology for dynamic characterization of NAT response. ADC maps at each observed imaging time point are converted to estimates of tumor cellularity. Spatial proliferation maps between pairs of imaging time points are then estimated using the biophysical model with histograms used to obtain summary metrics.

A schematic of the biophysical parameter characterization framework is shown in Fig. 2. Initial cell number was assigned at each observed imaging time point based on ADC images as described, and the forward model was used to estimate a region-based spatially varying proliferation rate map and global cell diffusion parameter. Model parameters were estimated using a quasi-Newton optimization method using L-BFGS^40^ with gradients for proliferation calculated using a numerically-efficient adjoint state method^41^ and gradients for diffusion calculated using a forward finite difference method with perturbation of 1%. Following parameter estimation, histograms of the tumor proliferation rate map were created with bin widths of 0.1 days^-1^. Histogram summary metrics of mean, median, interquartile range (IQR), standard deviation, 25th percentile, and the 75th percentile of tumor proliferation rate were recorded. We assume a piecewise continuous antitumor effect between each observed imaging time point during therapy and characterize phenotypic biophysical parameters between time point combinations: T_01_ (T_0_ and T_1_), T_02_ (T_0_ and T_2_), and T_12_ (T_1_ and T_2_). The proliferation maps are then interpreted using histogram analysis and assessed with histogram summary metrics. This allows for capture of the dynamic changes in parameters between time points as well as evaluation of the importance of intermediate time point imaging acquisitions.

We also compared our model-based analysis to conventional morphometric analysis metrics. We assessed the change in metrics between time point combinations: T_01_ (T_0_ and T_1_), T_02_ (T_0_ and T_2_), and T_12_ (T_1_ and T_2_) for the tumor longest dimension, mean tumor ADC^42^, and functional tumor volume (FTV)^43,44^.

Statistical analyses were performed to characterize the correlation of histogram summary metrics of tumor response (histogram mean, median and 75th percentile) with pathological response in both total and “in-breast” RCB. Total RCB was calculated through the web-based calculator provided by MD Anderson^45^ using tumor size, tumor cellularity, number of positive ALNs, and the diameter of the largest metastasis^46^, whereas in-breast RCB included only the tumor size and cancer cell density components of the RCB measurement, as previously suggested by Hylton et al^15^. In previous work, we used AUC of the receiver operating characteristic (ROC) curves to test our method’s ability to predict pCR. However, in this work we utilize a continuous outcome metric, RCB, with Pearson correlation coefficients and associated p-values were calculated to assess the degree of correlation between individual histogram summary metrics and both total and in-breast RCB.

## Results

**Figure 3:**
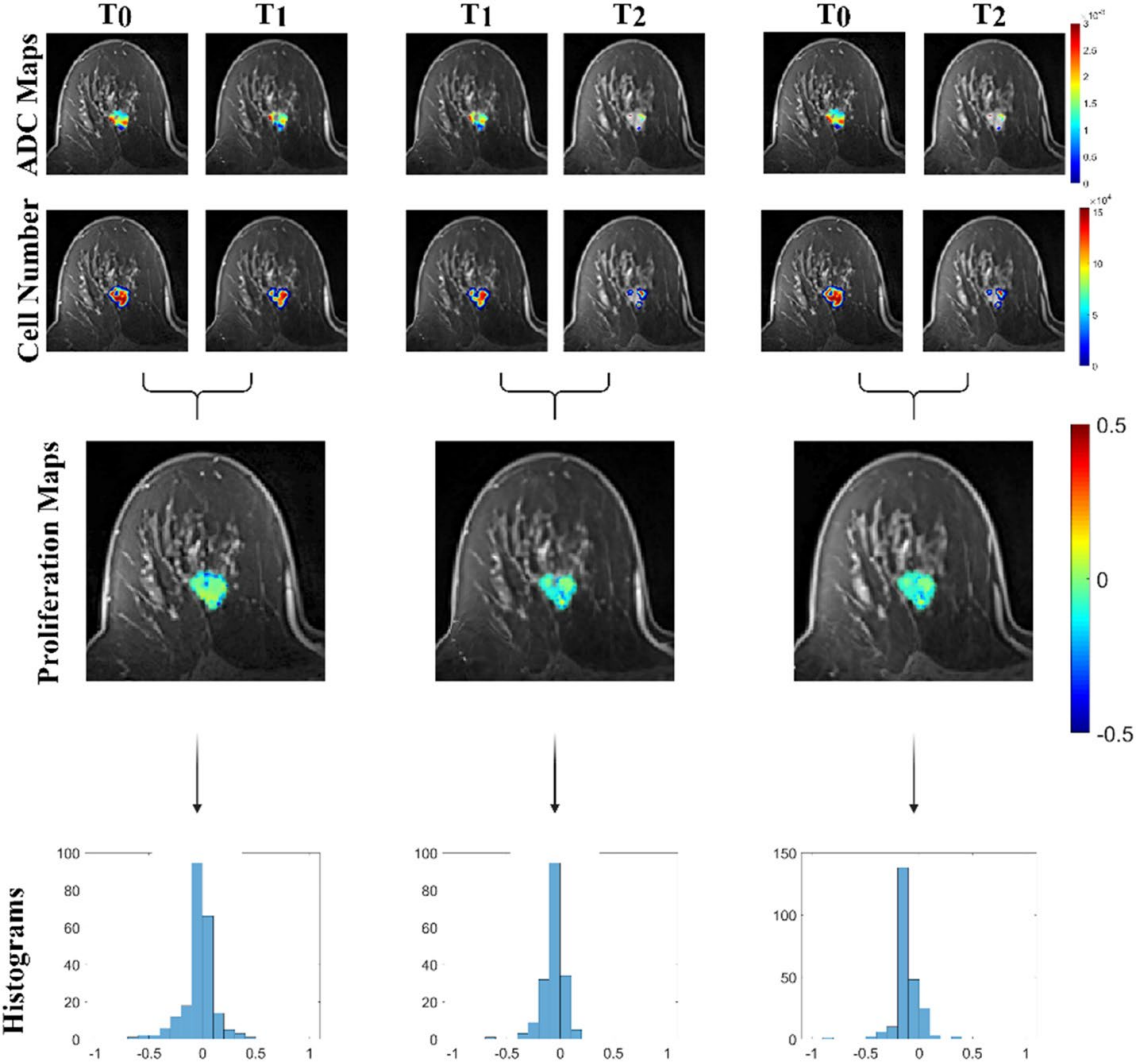
Representative contrast enhanced MR images overlaid with ADC maps, cell number estimates, proliferation maps, and proliferation histograms are shown for time point combinations from a patient with non-pCR with a RCB value of 1.94. This patient exhibited initial response at early time points with a robust net-negative tumor proliferation histogram.

**Figure 4:**
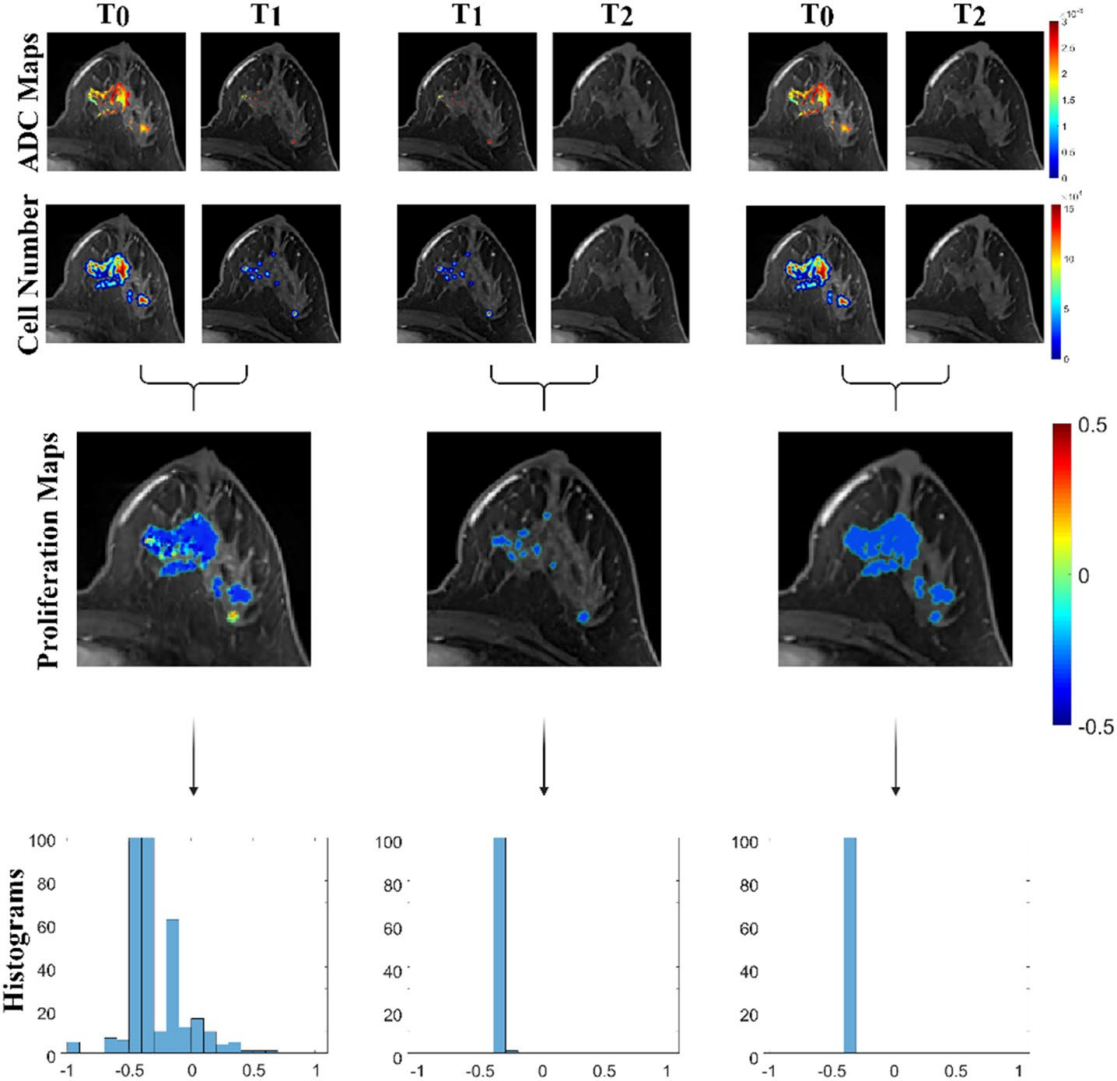
Representative contrast enhanced MR images overlaid with ADC maps, cell number estimates, proliferation maps, and proliferation histograms are shown for time point combinations from a patient with pCR (RCB 0). This patient exhibited robust initial response with full response by mid-treatment (T_2_).

A biophysical model of tumor growth and response is used to obtain parameter estimates of global diffusion and spatial proliferation rate based on changes of the observed cellularity between imaging time points, capturing patient-specific dynamic response during breast cancer NAT. We used histogram summary metrics characterizing the tumor spatial proliferation rate to test correlation with RCB. Representative imaging data and estimated biophysical parameter maps for tumors from patients with residual tumor burden and pCR outcomes are shown in Figs. 3 and 4, respectively. Qualitatively, the proliferation map results show that the representative non-responsive tumor exhibited an initial response to the Paclitaxel ± experimental drug treatment that ultimately progressed during the anthracycline portion of therapy. The proliferation map between T_0_ and T_2_ shows that the majority of the tumor responds to early therapy (blue), however there still exists regions of resistance with net tumor cell proliferation (yellow). For the representative responsive tumor, the proliferation map results show robust initial response to the Paclitaxel ± experimental drug treatment with no residual proliferative tumor during the anthracycline portion of therapy. The proliferation maps for this patient showed strong response (blue). Combined, representative images, proliferation maps, and histograms depict response to therapy for a responding patient, and progression of disease for a non-responding patient.

**Figure 5:**
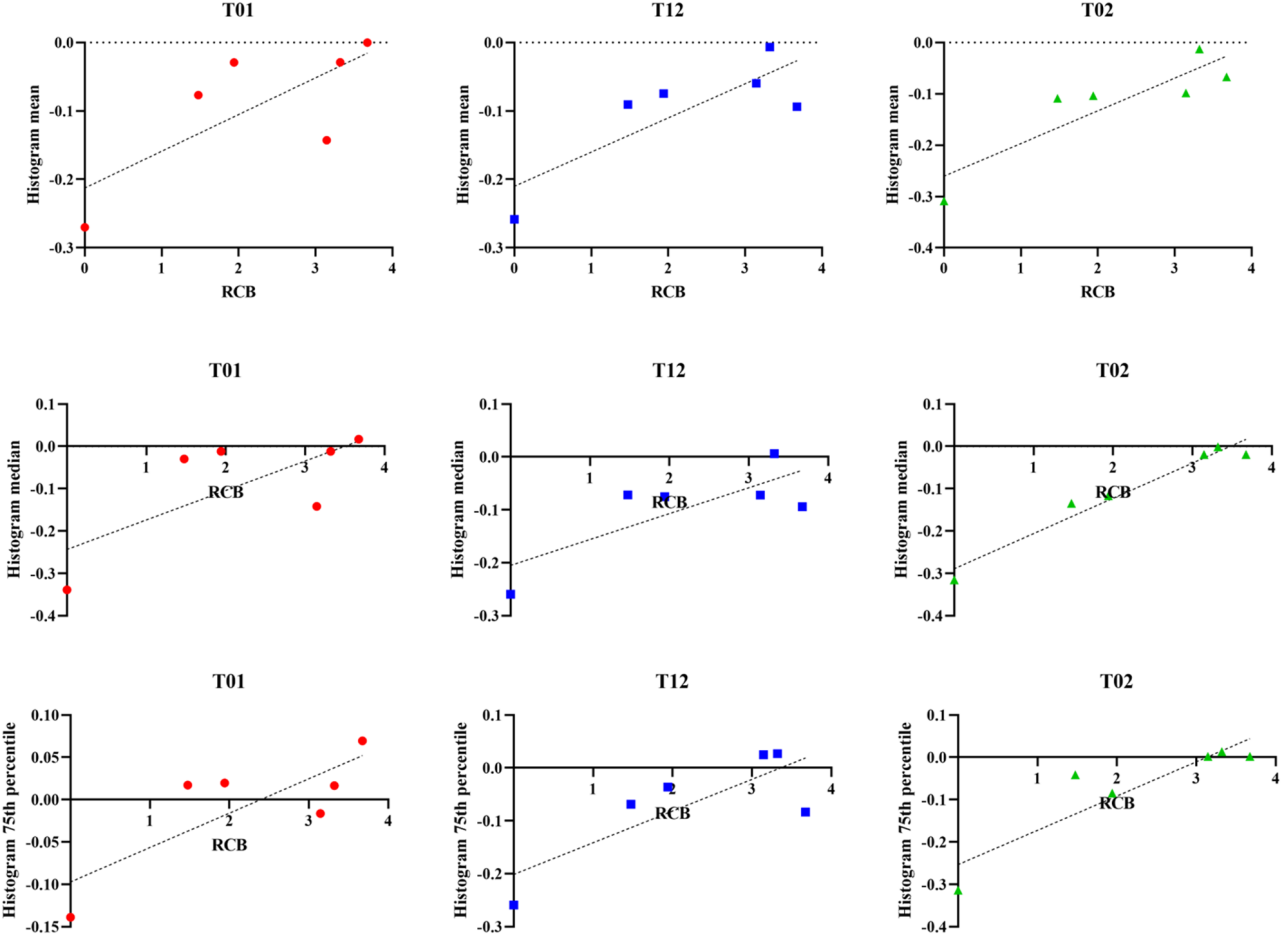
Plots of proliferation map histogram metrics for mean, median, and 75th percentile versus RCB. Spatial proliferation maps for each combination of observed time points were estimated using our model-based analysis framework. Histogram summary metrics were extracted and assessed for correlation to therapy response, as described by RCB value.

**Figure 6:**
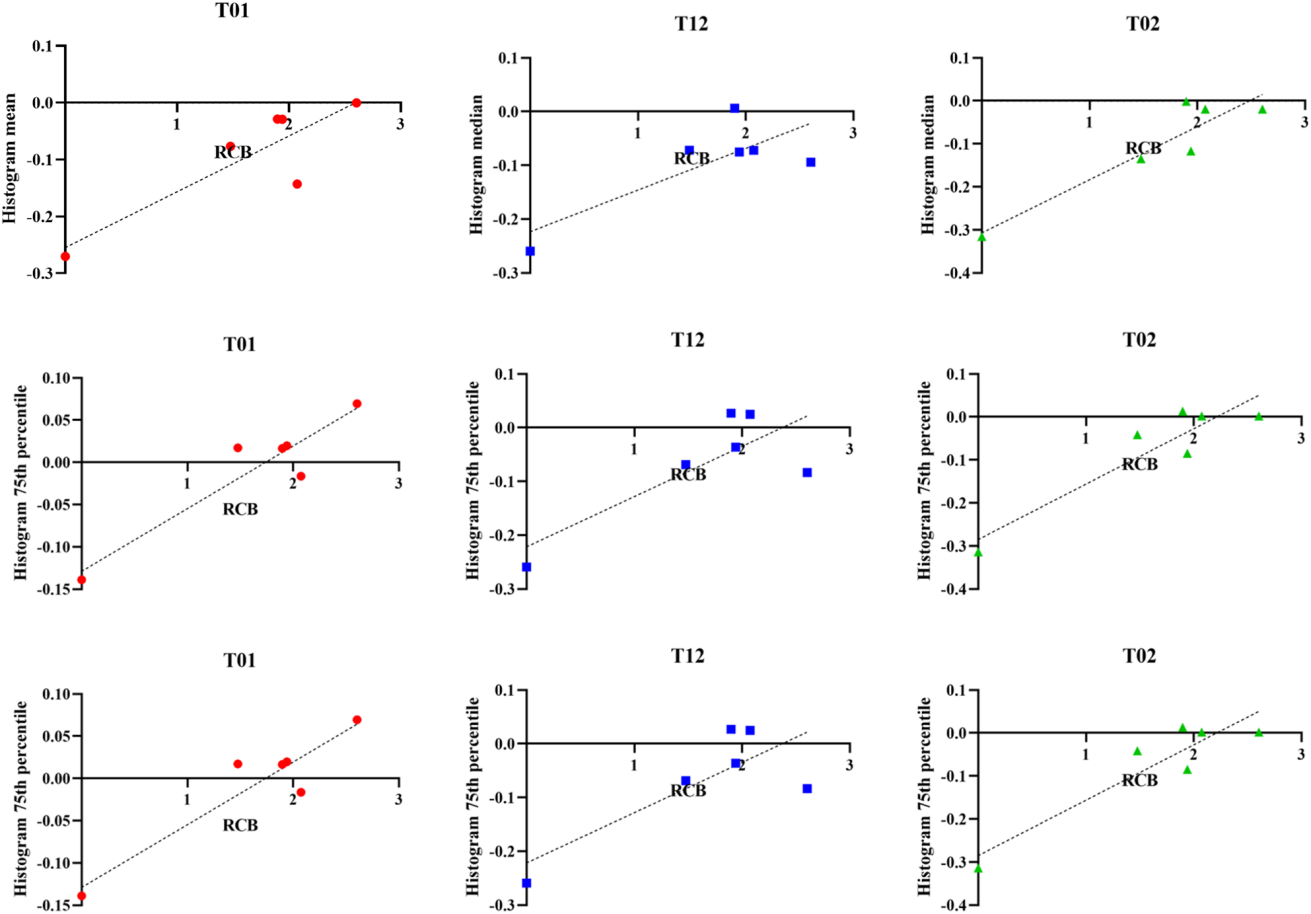
Plots of proliferation map histogram metrics for mean, median, and 75th percentile versus in-breast RCB. Spatial proliferation maps for each combination of observed time points were estimated using our model-based analysis framework. Histogram summary metrics were extracted and assessed for correlation to therapy response, as described by RCB value.

Correlation results for histogram summary metrics from proliferation maps at observed time point combinations with total RCB and in-breast RCB are shown in Figs. 5 and 6, respectively. Qualitatively, early time point analysis shows moderate to strong positive correlations between proliferation histogram metrics and RCB. Incorporation of additional imaging data with observations at later time points leads to an improved relationship. Model fits using data from T_0_ and T_2_ (T_02_) have a very strong uphill linear relationship between calculated histogram metrics and RCB. Table 2 reports the Pearson correlation coefficients, *r*, for each histogram metric with each time point combination for both total and in-breast RCB. In general, histogram metrics obtained from model fits using data from T_2_ (including T_1_ and T_2_, and T_0_ and T_2_) exhibit the strongest correlation to total RCB. Histogram metrics obtained from model fits between T_0_ and T_1_ exhibit the strongest correlation to in-breast RCB. The *p*-values for each proliferation histogram metric with each time point combination are reported in Table 3. As shown in Tables 2 and 3, our modeling methods are able to characterize the dynamic therapeutic response using the proliferation histogram metrics over the course of NAT.

**Table 2:** Pearson correlation coefficient for each proliferation histogram metric for each observed time point combination.

Metric	T_01_		T_12_		T_02_	
	Total RCB	In-breast RCB	Total RCB	In-breast RCB	Total RCB	In-breast RCB
Mean	0.7422	0.8682	0.8192	0.8244	0.8828	0.8896
Median	0.7176	0.8627	0.7754	0.7856	0.9764	0.9278
75th percentile	0.7994	0.9343	0.7945	0.7930	0.9049	0.9213
Change in longest dimension	0.4763	0.4995	0.1318	0.1128	0.3853	0.3876
ΔMean ADC	− 0.7605	− 0.7730	0.07662	− 0.0121	0.07971	0.2239
ΔFTV	0.1264	− 0.0064	− 0.3923	− 0.3107	− 0.4424	− 0.4273

**Table 3:** *p*-values for each proliferation histogram metric between each model observed time point pairs.

Metric	T_01_		T_12_		T_02_	
	Total RCB	In-breast RCB	Total RCB	In-breast RCB	Total RCB	In-breast RCB
Mean	0.0912	0.0249*	0.0461*	0.0436*	0.0198*	0.0176*
Median	0.1083	0.0270*	0.0700	0.0640	0.0008**	0.0076**
75th percentile	0.0563	0.0063**	0.0590	0.0598	0.0131*	0.0090**
Change in longest dimension	0.4959	0.2537	0.6858	0.8098	0.8317	0.3903
ΔMean ADC	0.0792	0.0714	0.8703	0.9795	0.8807	0.6697
ΔFTV	0.8114	0.9903	0.4417	0.5489	0.3797	0.3981

*Indicates *p* < 0.05 and ** indicates *p* < 0.01.

We also compared our model-based analysis to conventional morphometric analysis of change in longest dimension as well as change in mean ADC and change in functional tumor volume (FTV). Tables 2 and 3 show our proliferation histogram summary metrics of mean, median and 75^th^ percentile exhibit better correlation to RCB than conventional assessment methods. We found that change in longest dimension and change in FTV had poor to fair correlation with both total and in-breast RCB. The change in mean ADC between T_0_ and T_1_ has a moderate negative correlation to both total and in-breast RCB, but degrades in correlation with response at later time point assessments that include T_2_.

## Discussion

In previous work, we introduced a mathematical framework for predicting pathological response to NAT in breast cancer using imaging data acquired prior to the beginning of therapy and after one cycle of therapy^25^. In this work, we show proof-of-concept for a related approach within the I-SPY 2 imaging data acquisition framework to use biophysical modeling methods to interpret serial quantitative MR imaging data to characterize dynamic changes in response over the course of therapy. In this retrospective study, we show correlation of proliferation rate metrics with pathological outcomes at the time of surgery. Our approach is able to capture the dynamic, patient-specific response to NAT with significant correlation of proliferation histogram summary metrics to RCB that outperforms conventional morphometric analyses. In this work, we show that additional imaging acquisitions improve response characterization. This work also demonstrates proof-of-concept for potential real-time biophysical response assessment with promise to enable interventional therapeutic decisions during therapy, allowing the opportunity to tailor therapy based on patient-specific observed response. Following validation in future large-scale studies, these methods may offer opportunities to guide adaptive therapeutic regimens with a goal of increasing response and minimizing patient exposure to ineffective therapies.

This work in developing quantitative analysis tools for characterizing dynamic response to breast cancer NAT based on imaging data acquired during therapy is an important step towards realizing the eventual goals of personalized therapy selection and dynamic adaptive patient-specific therapy regimens. As shown in the representative example in Fig. 4, a patient with a responsive tumor potentially underwent additional anthracycline-based therapy already having demonstrated robust response with a lack of residual proliferative areas of tumor growth, based on our characterization. Anthracycline therapies are highly toxic and associated with significant survivorship concerns, including cardiotoxicity^47^ and secondary leukemia^48^. With the development of new tools able to characterize the dynamic biophysics of tumor response in patients that exhibit early response, options for treatment de-escalation may potentially spare adverse side effects associated with additional and/or unnecessary cytotoxic therapies. With respect to the representative patient with a non-responsive tumor, our characterization shows a highly heterogeneous proliferative treatment response, with potential isolated regions of drug resistance. While the majority of the tumor responds favorably with net-negative proliferation values, several isolated locations of positive proliferation remain prior to the start of anthracycline therapy. With the development of new analysis tools capable of characterizing these possible areas of resistance, there is potential for patient-specific optimization of therapy to increase the patient’s chance of achieving response. Additionally, combination of our spatial response characterization methods with imaging-guided biopsies may offer enhanced opportunity for personalized intervention with assessment of localized regions of treatment resistance. Including histopathological and/or genomics/proteomics analyses of these biopsies alongside our imaging-based model assessment using hybrid machine learning approaches may offer significant additional insight into the characterization of treatment response in this patient setting^49^.

While the results from this study are promising, they are not without several important limitations. First, we are using a small, single-site patient cohort as an initial proof-of-concept for our dynamic biophysical characterization methods. In future work, we will expand this analysis to include a larger multi-site cohort of imaging data to more robustly investigate the association of our model-based metrics with NAT response, including assessment within specific molecular subgroups of breast cancer. Second, in this work we use a slab analysis approach in which we assume the central tumor slice is representative of the entire tumor and use single-slice MR data with slice thickness of 1 mm to estimate biophysical parameters constrained to a two-dimensional mathematical model. While we have previously shown extension of a similar approach to three-dimensional analysis^1^, it is important to note that current computational demands for volumetric parameter estimation in full volumetric analysis limit throughput and clinical translational potential. It will be important to investigate computational speed enhancements through high performance computing and machine learning approaches in future work. Finally, while our approach currently captures first-order biophysical phenotypic phenomena of tumor cell proliferation and diffusive motility, our approach does ignore other secondary biological factors. However, our data-driven approach, while simple, is able to characterize important first-order dynamic biophysical factors to offer mechanistic interpretation of available patient-specific imaging data. However, despite these limitations our methods allow for mechanistic characterization of dynamic biophysical changes throughout the course of NAT with high correlation to residual disease, improving upon current conventional morphometric assessment methods.

Our methods characterize primary breast tumor response, consistent with many other breast NAT analysis methods, and ignores response within the axilla. Studies that use in-breast response assessment metrics to correlate with post-NAT staging systems that describe both primary and ALN response frequent the literature on imaging-based assessment. However, for an accurate/complete analysis, it is critical to examine outcome metrics that complement the observed assessment metrics, such as methods comparing primary tumor response to in-breast RCB, as done in this work. Clinical identification of therapeutic response includes both primary tumor response as well as nodal status, therefore thorough prediction of response should include assessment of both the primary tumor and ALN metastases. ALN metastasis is important in predicting overall recurrence and survival in breast cancer patients^50,51^. Additionally, NAT offers opportunities for less invasive and more conservative surgery through avoidance of ALN dissection, potentially sparing the patient from associated risks of morbidity, pain, neuropathy, limited arm abduction, lymphedema, and increased risk of cellulitis^52,53^. However, accurate methods for image-based characterization of ALN status are currently significantly limited^54^. Lymph nodes rapidly enhance with imaging contrast agents, complicating identification of malignancy. Current ALN radiological assessment methods are based on qualitative morphological characteristics that have limited predictive value. Further studies will be necessary to identify quantitative assessment methods capable of assessing and characterizing ALN status.

To summarize, we demonstrate that a model-based analysis can characterize biophysical metrics of spatial proliferation to capture dynamic changes in therapeutic response throughout the course of breast cancer NAT using quantitative imaging data. Our results show strong correlation of model-based metrics during the course of therapy with pathological response observations at the conclusion of therapy. Our data suggests that imaging-based biophysical modeling approaches have important potential to interpret and characterize serial imaging data acquired during NAT. This framework has the potential to advance the development of patient-specific response-adaptive therapeutic regimens whereby regimens with patient-specific doses and cycles of anti-neoplastic therapy can be optimized based on mechanistic observations of dynamic response to therapy. Future work includes expanding to a larger, multi-site patient cohort to rigorously validate our methods.

## Data Availability

The datasets used and/or analyzed during the current study available from the corresponding author on reasonable request.
